# Supplementary material for: Interventions for Child Drowning Reduction in the Indian Sundarbans: Perspectives from the Ground
Source: Children (Basel). 2020 Dec 14;7(12):291. doi: 10.3390/children7120291 (PMC7765013; doi:10.3390/children7120291)
Supplement: Supplementary file 1 [file children-07-00291-s001.pdf]

Supplementary:

Table S1. Consolidated criteria for reporting qualitative studies (COREQ): 32-item checklist.

No. Item	Guide Questions/Description	Reported on Page #
**Domain 1: research team and reflexivity**		
Personal characteristics		
1. Interviewer/facilitator	Which author/s conducted the interview or focus group?	2
2. Credentials	What were the researcher's credentials? e.g., PhD, MD	2
3. Occupation	What was their occupation at the time of the study?	2
4. Gender	Was the researcher male or female?	2
5. Experience and training	What experience or training did the researcher have?	2
Relationship with participants		
6. Relationship established	Was a relationship established prior to study commencement?	3
7. Participant knowledge of the interviewer	What did the participants know about the researcher? e.g., personal goals, reasons for doing the research	N/A
8. Interviewer characteristics	What characteristics were reported about the interviewer/facilitator? e.g., bias, assumptions, reasons and interests in the research topic	N/A
**Domain 2: study design**		
Theoretical framework		
9. Methodological orientation and theory	What methodological orientation was stated to underpin the study? e.g., grounded theory, discourse analysis, ethnography, phenomenology, content analysis	4
Participant selection		7–9
10. Sampling	How were participants selected? e.g., purposive, convenience, consecutive, snowball	3-4
11. Method of approach	How were participants approached? e.g., face to face, telephone, mail, email	3-4
12. Sample size	How many participants were in the study?	3-4
13. Non-participation	How many people refused to participate or dropped out? Reasons?	4
Setting		
14. Setting of data collection	Where were the data collected? e.g., home, clinic, workplace	3
15. Presence of non-participants	Was anyone else present besides the participants and researchers?	3
16. Description of sample	What are the important characteristics of the sample? e.g., demographic data, date	3-4
Data collection		
17. Interview guide	Were questions, prompts, guides provided by the authors? Was it pilot tested?	4
18. Repeat interviews	Were repeat interviews carried out? If yes, how many?	3
19. Audio/visual recording	Did the research use audio or visual recording to collect the data?	3
20. Field notes	Were field notes made during and/or after the interview or focus group?	3
21. Duration	What was the duration of the interviews or focus group?	3
22. Data saturation	Was data saturation discussed?	3
23. Transcripts returned	Were transcripts returned to participants for comment and/or correction?	3
**Domain 3: analysis and findings**		
Data analysis		
24. Number of data coders	How many data coders coded the data?	4

25. Description of the coding tree	Did the authors provide a description of the coding tree?	N/A
26. Derivation of themes	Were themes identified in advance or derived from the data?	4
27. Software	What software, if applicable, was used to manage the data?	4
28. Participant checking	Did participants provide feedback on the findings?	11
Reporting		
29. Quotations presented	Were participant quotations presented to illustrate the themes/findings? Was each quotation identified? e.g., participant number	Table S2
30. Data and findings consistent	Was there consistency between the data presented and the findings?	4-10
31. Clarity of major themes	Were major themes clearly presented in the findings?	4-10
32. Clarity of minor themes	Is there a description of diverse cases or discussion of minor themes?	4-10

Developed from: Tong A, Sainsbury P, Craig J. Consolidated criteria for reporting qualitative research (COREQ): a 32-item checklist for interviews and focus groups. *International Journal for Quality in Health Care*. 2007. Volume 19, Number 6: pp. 349–357.

Table S2. Illustrative quotations by theme.

Main Finding/ Theme	Ref.	Quote
Considerations for program design and delivery		
Acceptability across all interventions	1	"For one and half to two years, barriers are a good method, and next is the childcare one." (Community member, female, IDI)
	2	"Also, when he/she is a little grown up, they should be sent for swimming training. So, all three methods are necessary for different ages." (Community member, male, FGD)
Affordability	3	"Fencing is a one-time investment. If some organization pays a part of it, I can contribute the rest for that. But this one (childcare) is a recurring expenditure. That is not possible." (Community member, male, IDI)
Community engagement and ownership	4	"We are conscious [about issues] so long the meetings continue. After that it is back to square one." (Community member, male, FGD)
	5	You have to ignite that process through which they [the community] can identify the resources they have within and they can start taking action, because outside implementation and outside output doesn't sustain forever." (Grassroots NGO, IDI)
	6	"If you are putting up a fencing or a play pan type thing in my house, I will look after it. But as an organisation you also need to visit the place of installation on a regular basis to check its condition." (Community leader, IDI)
Resources and skill set	7	"One of the major reasons [for difficulty in implementing programs] is the geography, because these are very small islands and access to a main city is very difficult." (Grassroots NGO, IDI)
	8	"See, the first challenge with the program would be human resources, which are difficult to recruit and retain. People change jobs, which is natural, for so many reasons. Everybody wants to grow in life. Of course, packages and benefits are also other reasons." (Grassroots NGO, IDI)
Social class	9	"We didn't have proper space to set up the centre, and we were operating from someone's house. At that time when looking for a house, there were questions like, "How can a Muslim lady use my kitchen?"" (Anganwadi worker, IDI)
	10	"But there may be political problems. The political parties might interfere as they are involved in every aspect of our lives. Today I am associated with Trinamool. My friend who was with me, might join BJP and we may not talk anymore." (ASHA worker, IDI)
	11	"Poverty is more among the Muslims compared to Hindus...they might not pay" (Community leader, IDI)
Intervention-specific considerations		

Home-based barriers	12	"Actually, providing a barrier is a good concept. Putting up barriers on the veranda or courtyard is essential. Earlier, we also grew up with these." (ASHA worker, IDI)
	13	"For every single job we use pond water except drinking. When so many people are accessing the pond so many times a day it is natural that sometimes the barrier will not be closed properly." (Community member, male, FGD)
	14	"You have to provide them free space to move around [in the playpen] which is healthy for their growth." (Grassroots NGO, IDI)
	15	"If someone is with the child when the child is playing or doing something else inside the fence, we can be tension free. But under no circumstances the child can be kept alone without any attention inside the fence." (Community member, female, IDI)
	16	"We need to see the safety of the child but also keep the barrier a little short so that the adults don't face any problem while crossing over it." (Community leader, IDI)
	17	**"**If the family members want to build a barrier, it may not be according to plan. But if there is a properly trained person for this job, then the work will be of good quality." (ASHA worker, IDI)
Childcare and supervision-based programs	18	"Why won't they send their kids [to childcare services]. The kids will learn a few things and they will be safe." (Community member, female, IDI)
	19	"Each child will have different activities – one might be hungry, other might want to go for bathroom, someone might be thirsty. How can one person look after so many requirements?" (Community member, male, IDI)
	20	"There are few people who send their children [to ICDS centres] ... Most of them have no time. They have to drop their children as well as bring their children back home." (Community member, male, FGD)
	21	"She [a local woman] requires training. It is not guaranteed that a qualified woman can do everything. She should know how to control children, what to do, what not to etc." (Community leader, FGD)
	22	"The children will play. They might have some creative learning activities like storytelling etc. Basically, the aim should be to keep the children happy and engaged." (Community leader, IDI)
	23	"If it is a closed space, then mothers will be tension free." (Community member, female, FGDs)
	24	"But it is of no use [to only have a morning session]. Mother again goes out in the afternoon and gets busy. The children again go out to play and fall into the water." (Community member, female, IDI)
Swim and rescue training	25	"Everybody is not able to teach their children to swim. The parents leave for their work after sending their children to school. Mothers do not have time to teach." (Community member, female, FGD)
	26	"Local ponds are not suitable for training purposes. First local ponds are too deep. They are very dirty also." (Community member, male, IDI)
	27	"We need some first aid arrangement." (Community member, female, IDI)
	28	"Well they need a rest room or changing room. They can't directly jump into water coming from home." (Community member, female, IDI)
	29	"I prefer an outside trainer. This will give a better impression on the villagers as they will give more importance to an outside trainer rather than a local villager." (Community member, male, FGD)
	30	"If the road condition is not good, then how can a trainer from outside come to the village every day?" (Community member, female, IDI)
First responder program	31	"These tactics are not known to everybody. When the child falls in water, the trained person rescues the child and tries to bring water out of the body through this process. That would be good." (Community member, female, FGD)
	32	"There was a quack (local village doctor). He tried saving the child by spinning the child over his head. I insisted on taking him to the hospital at Darknagar. They reached hospital but by that time the child died…nobody listened to me. The child could have been saved if there was no wastage of time." (ASHA, IDI)

Other indigenous interventions for child safety	33	"If we can increase the awareness among the parents that would be good. People here are not so aware and have a careless attitude, that's why accidents happen." (Self-help group member, FGD)
	34	"In that case when the mother goes out for work, they tie up the child with rope in the house." (Anganwadi worker, IDI)
Use of government programs in drowning intervention delivery		
ASHA workers	35	"Yes, we can add them [barrier maintenance] with our child care services." (ASHA worker, IDI)
	36	"We cannot devote so much time as we always have to rush to emergencies if a mother falls ill, or the hospital administration summons us." (ASHA worker, IDI)
	37	"Every work has some financial implications. We work the whole night just to earn some money. I don't want to say anything on your face but I expect that you will definitely pay something for getting this type of work done." (ASHA worker, IDI)
Self-help groups	38	"I will give the responsibility of each crèche to 2 to 3 members. This responsibility will be rotated among other members on a rotational basis. Each member will take responsibility to the time which is suitable for them." (SHG member, FGD)
	39	"We will go from door to door and convince mothers to put barriers as there are water bodies around." (SHG member, FGD)
	40	"I don't think anyone in this group can be directly involved and give time. Everyone is involved with their own businesses." (SHG member, FGD)
	41	"Self-help groups are less challenging to engage. It is better for delivering programs in the community by giving small incentives. Fewer permissions and formalities are required." (Grassroots NGO, IDI)
Anganwadi centres (ICDS program)	42	"Respondent 4: Yes, some education is provided.<br>Respondent 5: Not much education is provided. Basically, the kids go and stay there that's all.<br>Respondent 1: In other places, they do teach the kids but not here." (Community member, female, FGD)
	43	"The children were holding the books but there was no focus… Mothers were very talkative and there was absolute chaos in the centre…The teacher was confused, and there was no set routine or instructions from her." (Anganwadi centre observation)
	44	"There was no room. The students assembled under a tree on a wooden cot covered by tarp overhead." (Anganwadi centre, 0bservation)
	45	"We are working 4 to 5 hours a day in the centre. After that every day till 12 noon we need to do lot of entries in the government register…Beside these, we need to go to the field every day." (Anganwadi worker, IDI)
	46	"It's been nearly 3 years that the helper has left. Since then I have not been provided with any helper." (Anganwadi worker, IDI)
	47	"One member from Gram Panchayat formed a committee in our area for these kinds of jobs [overseeing ICDS]. The ICDS worker and ASHA worker hold meetings with them. But these kinds of committees are not run in all areas." (community member, male, FGD)
	48	"Insects in rice, cereals, everywhere. Some days they give half an egg, some days no egg at all. Why should parents send their children to the centre? Sometimes, the children fall sick after eating such unhygienic food." (SHG member, FDG)
	49	"If you held a meeting in a block, there are ICDS program distributors at the block level. In the meeting, most of the distributors don't come … if there is order from their own department then they will come." (Grassroots NGO, IDI)
	50	"So it was challenging [engaging the government to improve Anganwadi centres]. There were some queries from the departments that if the childcare centre is run by an NGO…if anything happens in the centre, then who will be responsible?... They were in a dilemma about what should be done. They still haven't decided, it will take time." (Grassroots NGO, IDI)

	51	“They stay in Anganwadi school from 7 a.m. to 9:30 a.m. Then they move to NGO school where they stay from 11 a.m. to 1:30 p.m.” (Community member, female, FGD)
Other community programs	52	“Yes, initially the NGO should run the programme with the help of local clubs. Whenever you are taking the local clubs into confidence, you will always find 5 to 7 youths ready to help you all time… Slowly you hand over the work to the local club.” (Community leader, IDI)
Stakeholder analysis		
Block-level officials	53	“It will be effective if you contact the Block Development Office. He controls every Gram Panchayat. If you let him know he will alert every Panchayat leader. Then you contact the respective Panchayat leader where you want to launch this program.” (Community leader, FGD)
	54	“You should approach the Panchayat first and discuss in detail about the programme. Panchayat can help you find a suitable pond where you can start the programme.” (Community leader, IDI)
	55	“The administrators create problems - the Panchayat and his team. If the government launches any scheme, they are ones who create problems … Suppose the government is making arrangements for the ICDS program, these people would come and say that the program should run at their place.” (Community member, female, FGD)
Community leaders	56	“Village Head, cluster Female Leaders…For any work they need to be informed first. We discuss with them before taking up any work.’ (SHG member, IDI)
Local police stations	57	“Also, you need to inform the local police station. They need to be in loop in case any accident happens.” (Community leaders, FGD)
Community members	58	“No. You need to discuss with the Villagers also. You need to describe the entire programme to them. Discuss all the services that you will give to the people. The villagers will give input on the location of the centre and how to go about the programme.” (Anganwadi worker, IDI)
